# Time-Course Transcriptome Analysis of the Lungs of Mice Challenged with Aerosols of Methicillin-Resistant *Staphylococcus aureus* USA300 Clone Reveals Inflammatory Balance

**DOI:** 10.3390/biom13020347

**Published:** 2023-02-10

**Authors:** Yue Zhao, Lina Zhai, Tongtong Qin, Lingfei Hu, Jiazhen Wang, Zhijun Zhang, Chengyu Sui, Lili Zhang, Dongsheng Zhou, Meng Lv, Wenhui Yang

**Affiliations:** 1 Department of Immunology of Basic Medical College, Guizhou Medical University, Guiyang 550025, China; 2 State Key Laboratory of Pathogen and Biosecurity, Beijing Institute of Microbiology and Epidemiology, Beijing 100071, China; 3 College of Life Science and Technology, Beijing University of Chemical Technology, Beijing 100029, China

**Keywords:** methicillin-resistant *Staphylococcus aureus*, USA300, pneumonia, molecular pathology, time-course transcriptome, resolution of inflammation

## Abstract

USA300, a dominant clone of community-acquired methicillin-resistant *Staphylococcus aureus* (CA-MRSA), is circulating globally and can cause necrotizing pneumonia with high morbidity and mortality. To further reveal the host anti-MRSA infection immune response, we established a mouse model of acute primary MRSA pneumonia challenged with aerosols of the USA300 clone. A time-course transcriptome analysis of the lungs collected at 0, 12, 24, 48 and 96 h post-infection (hpi) was conducted using RNA sequencing (RNA-seq) and multiple bioinformatic analysis methods. The change trend of histopathology and five innate immune cell (neutrophils, mononuclear cells, eosinophils, macrophages, DC cells) proportions in the lungs after infection was also examined. We observed a distinct acute pulmonary recovery process. A rapid initiation period of inflammation was present at 12 hpi, during which the IL-17 pathway dominantly mediated inflammation and immune defense. The main stages of host inflammatory response occurred at 24 and 48 hpi, and the regulation of interferon activation and macrophage polarization played an important role in the control of inflammatory balance at this stage. At 96 hpi, cellular proliferation processes associated with host repair were observed, as well as adaptive immunity and complement system responses involving C1q molecules. More importantly, the data provide new insight into and identify potential functional genes involved in the checks and balances occurring between host anti-inflammatory and proinflammatory responses. To the best of our knowledge, this is the first study to investigate transcriptional responses throughout the inflammatory recovery process in the lungs after MRSA infection. Our study uncovers valuable research targets for key regulatory mechanisms underlying the pathogenesis of MRSA lung infections, which may help to develop novel treatment strategies for MRSA pneumonia.

## 1. Introduction

*Staphylococcus aureus* (SA) is the most frequent cause of lower respiratory tract infection. Over the last three decades, the morbidity and mortality of SA pneumonia have increased dramatically because of the emergence of methicillin-resistant *Staphylococcus aureus* (MRSA) [1,2,3]. MRSA, a superbug that accounts for 50% of SA pneumonia and results in mortality as high as 56% [4,5], is on the rise globally and has become one of the biggest public health threats since the development of antibiotic treatment [6,7]. Compared with hospital-acquired MRSA (HA-MRSA), community-acquired MRSA (CA-MRSA) can occur in healthy individuals unrelated to the medical environment [8], indicating that CA-MRSA strains possess greater virulence and transmissibility than traditional HA-MRSA strains. MRSA has been divided into 12 distinct genetic clusters, named USA100 to USA1200 [8]. The USA300 and USA400 clone are the main strains of CA-MRSA [9], but USA300 is the dominant causative pathogen, spreading more widely and accounting for 97% of all CA-MRSA infections [3,8,10]. Vancomycin and linezolid are currently the only antibiotics for treatment of MRSA pneumonia, but both are limited by side effects [11,12] and may aggravate the antimicrobial resistance. Even with these treatments, there is a high mortality rate [13]. In consequence, the increased antibiotic resistance situation has heightened research efforts in developing new and alternative control strategies for MRSA pneumonia.

The different outcomes of confrontations between the host immune system and infectious bacteria determine the course and outcome of a disease. Hence, understanding the host’s immune response is critical to developing new intervention strategies. The first line of defense during infection is regulated by the host’s innate immune response. As the key cells of the innate immune system, macrophages and neutrophils can effectively control the infection of MRSA. Neutrophils have been extensively studied because of their strong bactericidal ability in the defense against MRSA infection [14,15,16]. The loss of alveolar macrophages can inhibit the killing of lung bacteria and significantly increase the mortality rate [17,18]. However, MRSA secretes a variety of virulence factors, including but not limited to hemolysins, extracellular proteases, and leuko-cytochromes that help it evade host immune defenses [19]. Moreover, growing evidence indicates that SA has evolved to utilize host innate immune molecules to evade eradication. The absence of certain immune molecules can surprisingly improve survival in mice [20]. Therefore, in the battle between the host and MRSA, the key mechanism determining the final outcome remains unclear.

Based on the above, we used a highly virulent and multidrug-resistant strain that belongs to the MRSA USA300 clone to develop a mouse model of primary MRSA pneumonia, and then performed time-course RNA sequencing of lung tissues from the mice to reveal dynamic changes in the host lung immune system. This work can provide a deeper understanding of the interaction mechanisms of MRSA with the host immune system and provide a new target for nonantibiotic treatment of MRSA pneumonia.

## 2. Materials and Methods

### 2.1. Bacterial Strain and Growth Conditions

The ATCC BAA-1556 strain, which belongs to the USA300 clone and is resistant to a variety of antibiotics such as methicillin and tetracycline, is maintained in our laboratory. The strain was inoculated in brain heart infusion (BHI) broth (BD Biosciences, Lawrence, KS) and grown overnight at 37 °C in a shaking incubator at 220 rpm for 12–14 h. The overnight culture was diluted at 1:200 with BHI and maintained at 37 °C for 3.5 h at 220 rpm. Then, the culture was further diluted and incubated for another 3.5–4 h to achieve OD_600_ 1.8–2.0. After that, the culture was adjusted to OD_600_ 1.8 for subsequent use, a concentration of approximately 6 × 10^8^ CFU/mL.

### 2.2. Animal Infection and Sample Acquisition

Six-week-old female C57BL/6Cnc mice were purchased from Vital River Laboratories (Beijing, China) for use in experiments. The mice were housed under pathogen-free conditions with free access to food and water under a 12:12 h light:dark cycle. Before the experiment, all the mice were divided into five groups (0 h control group and 12 h, 24 h, 48 h and 96 h postinfection groups). Each group included six mice, three of which were used for pathological detection and RNA sequencing, and three of which were used for flow cytometry. Control group mice were administered 50 μL saline, and mice in the four infection groups were challenged with 50 μL USA300 (1 × 10^8^ CFU) via aerosolized intratracheal inoculation, as described before [21]. Briefly, mice were anesthetized by intraperitoneal injection of pentobarbital sodium at a weight of 70 mg/kg. The mice were then immobilized, and the bacterial suspension or saline was aerosolized by a Micro Sprayer (Huironghe Company, Beijing, China) and delivered into the lungs through the tracheal branch of the mice. Control group mice were euthanized immediately after inoculation as 0 h samples. Other groups of mice were euthanized at 12, 24, 48 and 96 h after lung infection. The lung tissues of three mice in each group were divided into two parts for pathological examination and RNA extraction and sequencing.

### 2.3. Histopathological Validation

The lung tissue specimens of mice were placed in 4% paraformaldehyde fixative solution, then the fixed tissues were sectioned, embedded in paraffin, and stained with hematoxylin–eosin (HE). A slice scanner, PANNORAMIC DESK/MIDI/250/1000 (3DHISTECH, BP, HU) was used for panoramic scanning and the supporting software CaseViewer 2.4 for browsing and screenshots.

### 2.4. RNA Extraction, Library Preparation, and Sequencing

Collected lungs were soaked in RNAlater stabilization solution (Invitrogen, Carlsbad, CA, USA), and total RNA was extracted using the purification kit RNAprep pure tissue kit (Tiangen, DP431, Beijing, China). For the RNA samples extracted, purity was determined by NanoPhotometer^®^ (IMPLEN, Westlake Village, CA, USA). At the same time, the assay kit 2100 RNA Nano 6000 (Agilent Technologies, Santa Clara, CA, USA) was used to measure concentration and integrity.

After purity and integrity tests were passed, the samples were sent to Easyresearch Company (Beijing, China) for sequencing and library construction. Different index tags were selected following the Illumina^®^ kit operation instructions to generate sequencing libraries. Then, V1.5 reagent was used to perform clustering and double-ended sequencing on the NovaSeq 6000 S4 platform to obtain 150 bp double-ended sequencing reads. To ensure data quality, the raw sequence must be filtered (removing excessive sequences containing sequenced connectors and low quality bases) to obtain a high-quality sequence (CleanData) that is then compared to the reference genome (mice) using HISAT2 (version 2.1.0). Finally, HTSeq (version 0.6.0) was used to calculate gene expression levels of all samples and to generate gene expression profile data in FPKM (fragments per kilobase million mapped reads) [22]. FPKM eliminates the effects of gene length and sequencing differences on gene expression [23].

### 2.5. Screening and Functional Analysis of DEGs

First, the distribution and repeatability of samples were examined by principal component analysis (PCA). Then, the edgeR package in R was used to standardize the gene expression levels of all samples and to screen for differential genes [24]. Triplicates of the RNA-seq experiments were analyzed separately and resulting *p*-values adjusted using the Benjamini–Hochberg method for controlling the false-discovery rate (FDR). Differentially expressed genes (DEGs) were defined based on a false-discovery rate (FDR) < 0.05 and log_2_|fold change| ≥ 1. Finally, the Clusterprofiler package in R was used for GO and KEGG enrichment analysis [25], with Benjamini–Hochberg again used to adjust the *p*-values. If the *P*_adj_ value were less than 0.05, gene expression was considered significantly enriched.

### 2.6. Time-Series Gene Clustering

The Mfuzz R package [26] was used for cluster analysis of gene dynamic expression patterns based on a fuzzy C-means algorithm [27]. The cluster number was set to 6 and the fuzzy coefficient M was set to 2.

### 2.7. Construction of WGCNA and Core Gene Extraction

Weighted gene coexpression network analysis (WGCNA) was performed on 15 mouse lung samples using the WGCNA package in R [28]. Modules with a high correlation with sample characteristics were selected for further analysis (GO and KEGG enrichment). At the same time, Cytoscape (version 3.9.1) was used to visualize the gene interaction network and detect core genes in the network [29].

### 2.8. Flow Cytometric Analysis

For a better understanding of the pathological mechanisms of disease, we used flow cytometry to detect immune cell changes at corresponding time points. Single cells of lung tissue were obtained by digestion, removal of erythrocytes with a red blood cell lysis buffer and washing in phosphate-buffered saline (PBS) with 2% bovine serum albumin. Cells were then counted with a Countess II FL automated counter (Thermo Fisher Scientific, Waltham, MA, USA). Approximately 2 × 10^6^ cells per mouse were incubated in blocking solution containing 0.25% FcBlock (catalog number 553141, BD Biosciences, San Jose, CA, USA) in PBS for 15 min and then stained with fluorochrome conjugating antibodies for 30 min at 4 °C. After staining, cells were washed and fixed with 1% paraformaldehyde in PBS for 30 min, and then washed and resuspended in PBS with 2% bovine serum albumin. Flow cytometry was conducted with a BD FACSymphony A5 flow cytometer using BD FACSDiva software (BD Biosciences, San Jose, CA, USA). The antibodies used were: fixable viability stain 510 (mouse, BD Biosciences, 564,406); anti-CD45 (mouse, BD Biosciences, 564,279); anti-Ly6c (mouse, BioLegend, 128,011; San Diego, CA, USA) anti-Ly6G (mouse, BioLegend, 127,641); anti-CD11b (mouse, BioLegend, 101,237); anti-CD11c (mouse, BioLegend, 117,308); anti-CD64 (mouse, BioLegend, 139,305); anti-CD206 (mouse, BioLegend, 141,727); anti-CD86 (mouse, BioLegend, 105,014); anti-mouse F4/80 (mouse, BioLegend, 123,116); anti-Siglec-F (mouse, BioLegend, 155,507); and anti-I-A/I-E (mouse, BioLegend, 107,602). Data were analyzed by FlowJo Version 10 (BD Biosciences, San Jose, CA, USA). Experiments were performed in three replicates. The specific flow cytometric classification methods are shown in Figure S1.

### 2.9. Protein Interaction Network Analysis

The STRING (search tool for the retrieval of interacting genes/proteins) database was used to reveal functional protein–protein interactions (PPIs) among the genes in each module clustered by WGCNA.

### 2.10. Contrast qRT-PCR with RNA-Seq

To verify the results of RNA-seq data, the expression patterns of 16 DEGs at five time points were randomly selected for qRT-PCR verification. qRT-PCR was performed using a 7500 real-time PCR instrument (Life Technologies Holdings Pte Ltd., Singapore) and a total volume of 20 μL per reaction, including 10 μL TransStart Tip Green qPCR SuperMix, 2.4 μL of upstream and downstream primers (5 μM), and 7.6 μL of cDNA template. The reaction conditions were set as: 95 °C for 180 s, followed by 40 cycles at 95 °C for 15 s and 60 °C for 30 s, followed by 72 °C for 30 s. Each reaction was set up in triplicate.

We used Primer Express (version 3.0.1) to design primers, shown in Table S1. The β-actin gene was used as the reference gene for data normalization, and the relative expression values of selected genes were calculated by the 2−ΔΔCt. method. Correlations between RNA-seq and qRT-PCR data are given as Pearson correlation coefficients.

### 2.11. Statistical Analysis

Data are represented as means ± SEM. Flow analysis data were analyzed using a one-way ANOVA with Bonferroni’s multiple comparison test. All *p* values less than 0.05 were considered significant. Statistical analysis was performed using Prism 8.0 (GraphPad Software, La Jolla, CA, USA).

## 3. Results

### 3.1. Observations of Symptoms and Histopathology

All mice in the control group were healthy and energetic, but mice infected with USA300 deteriorated over time and showed severe hemorrhagic pulmonary inflammation. At 12 h post-infection (hpi), the behavior of infected mice changed, including slowness of movement and shortness of breath. At 24 hpi, all infected mice exhibited depression, polypnea and disordered fur, indicating that the disease had worsened over time. At 48 hpi, the symptoms of tachypnea and slowness of movement had improved. At 96 hpi, the mice were mostly recovered, with no depression, and normal breathing.

**Figure 1:**
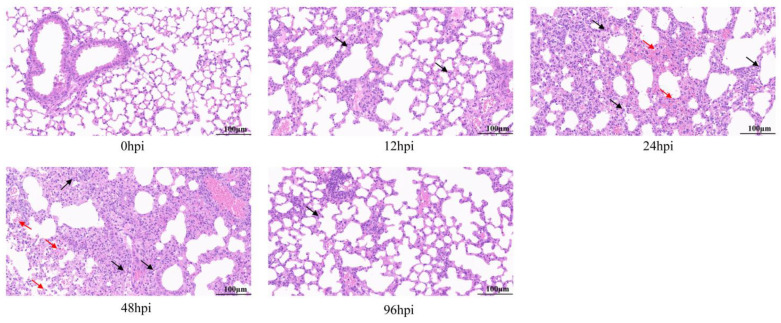
Pathological analysis of lung tissue infected with MRSA by hematoxylin and eosin (H&E) staining. Mice were challenged with USA300 strain (ATCC BAA-1556) of 1 × 10^8^ CFU, and lung pathological changes were observed at 12, 24, 48 and 96 h after infection. Arrows show lesions: inflammatory cell infiltration is shown in black and bleeding is shown in red (original magnification = 40×; scale bar = 100 μm). hpi = hours post-infection.

Histopathological analysis of the lungs by HE staining showed that all infected lung tissues had inflammatory symptoms (Figure 1). At 12 hpi, lymphocyte and neutrophil infiltration, partial alveolar wall thickening, and mild pulmonary edema were observed in lung tissues. At 24 and 48 hpi, lesions became worse, accompanied by more inflammatory cell infiltration, tissue bleeding and necrosis of epithelial cells. However, at 96 hpi, the degree of tissue lesion was reduced, bleeding and necrosis disappeared, inflammatory infiltration resolved, and most tissue structure had recovered.

Thus, lung inflammation and resolution occurred in mice within 96 hpi, suggesting a homeostasis repair process. In summary, we successfully established a mouse model of lung inflammation with USA300 infection and used it to obtain RNA-seq samples.

### 3.2. Identifications of Differential Genes and Enrichment Pathways

**Figure 2:**
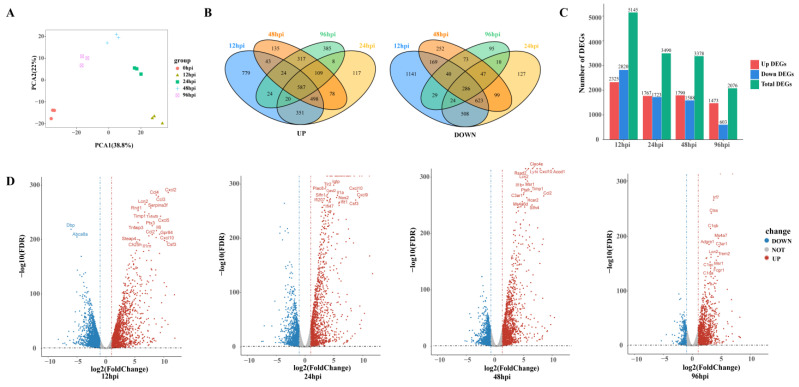
An overview of transcriptome analysis. (**A**) Principal component analysis (PCA) results of lung tissue RNA-seq data after SA infection. The same color represents replicates of the same stage. (**B**) Venn diagram comparing the upregulation and downregulation differentially expressed genes (DEGs). (**C**) Histograms show different genes at different time points. (**D**) Volcano plot of RNA-seq transcriptome data displaying the pattern of gene expression. Significant DEGs (FDR *p* ≤ 0.05) are highlighted in red (upregulated) or blue (downregulated). Curatorial genes with specific biological functions are labeled.

To detect transcriptional changes, we isolated and sequenced RNA from USA300-infected lung tissue to record gene expression profiles in a time-series fashion. Principal component analysis was used to assess the quality of data and the distribution of samples (Figure 2A). The first principal component (PCA1) accounted for 38.8% of the total expression differences in the first 1000 genes, and the second principal component (PCA2) accounted for 22%. Figure 2A reveals not only good reproducibility of replicate samples from the same time point, but also distinct transcriptional characteristics for samples from different time points.

A total of 6882 DEGs were identified across four MRSA infection time points. Compared with the control group, 695 and 265 genes were upregulated and downregulated, respectively, in all groups combined (Figure 2B). There were 5145 DEGs at 12 hpi, including 2325 upregulated genes and 2820 downregulated genes. By 96 hpi, the upregulated and downregulated genes had decreased to 1473 and 603, respectively (Figure 2C). The number of DEGs decreased over time, suggesting that the host has a repair process after lung infection.

At 12 hpi, most of the genes upregulated were related to promoting inflammatory response, such as chemokines (*Cxcl2*, *Ccl4*, *Cxcl5*, *Ccl2*, *Cxcl10*) and proinflammatory factors (*Il6*, *Tnfrsf9*, *Tnfaip3*, *Lcn2*), and genes related to the regulation of glucose metabolism and lipid metabolism *(Steap4*, *Ch25h*) were also upregulated at 24 hpi and 48 hpi, upregulated genes were mostly those involved in immune regulation, such as the activation and maturation of immune cells (*Slfn4*, *Cd274*, *Rsad2*, *Ms4a6d*, *Clec4e*). Complement-related genes (*C1qb*, *C3a1*, *C1qc*, *C1qa*) were significantly upregulated at 96 hpi (Figure 2D).

### 3.3. Analysis of Expression Patterns of DEGs

**Figure 3:**
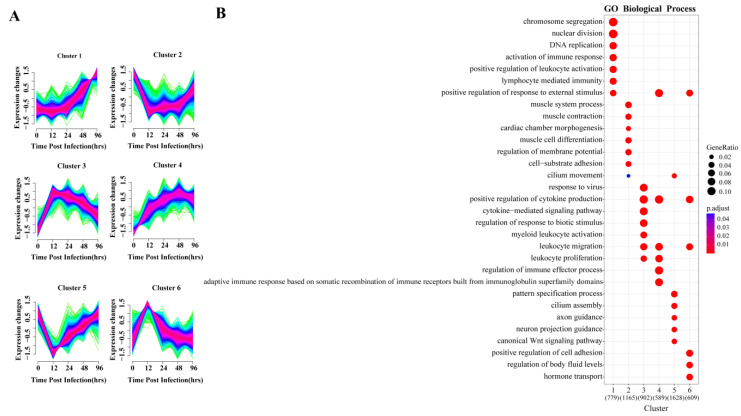
Cluster analysis of expression patterns of DEGS and related biological functions. (**A**) Mfuzz cluster analysis identified six different gene expression time patterns. The color varying gradually from green to red represents that the trends of genes become more suitable to the changes of the cluster. (**B**) Heat map showing the significance of the gene ontology (GO) terms in the biological processes describing each of the six clusters. The number of enriched genes belonging to each cluster is shown in parentheses.

To explore the dynamic changes in gene expression levels over time, the DEGs were divided into six gene clusters according to different expression patterns using Mfuzz, and the functional processes associated with each gene cluster were evaluated in a GO enrichment analysis (Figure 3). The identified processes were consistent with the molecular pathophysiology of disease progression.

The gene expression of clusters 2 and 5 decreased directly after infection and reached their lowest point by 12 hpi, and then slowly increased again. GO analysis indicated that the 1165 genes in cluster 2 were mainly involved in muscle metabolism, such as muscular system processes, muscle contraction and muscle cell differentiation. The 1628 genes of cluster 5 were enriched for normal physiological processes, including cilium assembly, WNT signaling pathway and pattern specification process. Studies have shown that the Wnt/β-catenin pathway plays an important role in alveolar development [30], indicating that the lung is damaged and normal development is inhibited at 12 hpi.

Gene expression levels in clusters 3, 4 and 6 began to increase immediately after infection. GO analysis revealed that 902 and 589 genes in clusters 3 and 4, respectively, were enriched in biological processes related to the regulation of immune response, including leukocyte activation and migration, positive regulation of cytokine production, cytokine-mediated signaling and cytokine secretion, respectively. In cluster 6, 609 genes were enriched for biological processes, including regulation of cell adhesion and body fluid levels, and hormone transport.

The gene expression of cluster 1 held steady initially, beginning to increase continuously after 24 hpi. GO enrichment indicated its 779 genes were mainly involved in two biological processes related to tissue repair and immune response. In the enrichment results, chromosome separation, nuclear division and DNA replication were all related to cell proliferation, which was closely related to tissue repair. Importantly, lymphocyte-mediated immunity was only enriched in this cluster, and lymphocytes are mostly associated with adaptive immune response.

Each gene cluster above had unique expression patterns and functions, but at the same time, gene clusters with similar early expression patterns had common enrichment pathways. The four upregulated gene clusters were mainly involved in the biological processes of immune and inflammatory responses, and two of them peaked at 12 hpi. The two downregulated gene clusters were related to biological processes of normal cell activities, and both decreased to lowest value at 12 hpi. In summary, 12 hpi is an important node of host transcriptional changes.

### 3.4. Network Analysis Identifies Functional Modules

**Figure 4:**
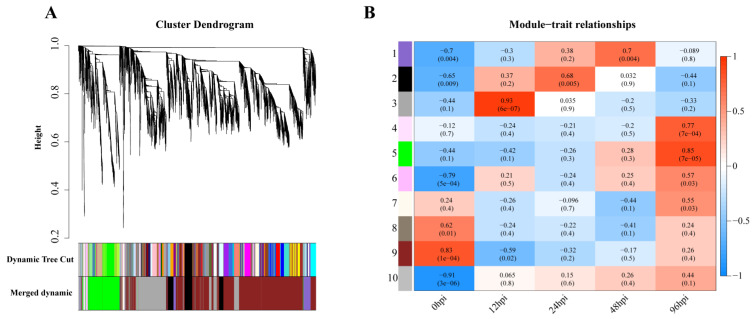
Gene modules in a weighted gene coexpression network analysis (WGCNA). (**A**) Hierarchical cluster trees (cluster trees) represent identified coexpressed gene modules at different stages of infection. Height (*Y*-axis) represents coexpression distance, and the *X*-axis corresponds to genes. In WGCNA convention, genes within different modules are labeled with different colors. (**B**). Heat map showing the relationship between modules and traits. Red indicates a positive correlation (0 < r < 1) between modules and infection stages, while blue indicates a negative correlation (−1 < r < 0).

Weighted gene coexpression analysis was carried out for the observed DEGs, and the dynamic tree-cutting algorithm in the WGCNA R package was used to process the hierarchical clustering tree. A total of 10 different modules were obtained. Different colors represent different modules (Figure 4A). Next, we calculated the correlation between the infection process (hpi) and modules, assigning modules random numbers from 1 to 10 (Figure 4B). Module 10 is the default module and includes discarded genes that cannot be aggregated. To evaluate the main function of each module’s genes, enrichment analysis was performed for modules with correlation coefficients greater than 0.5.

**Figure 5:**
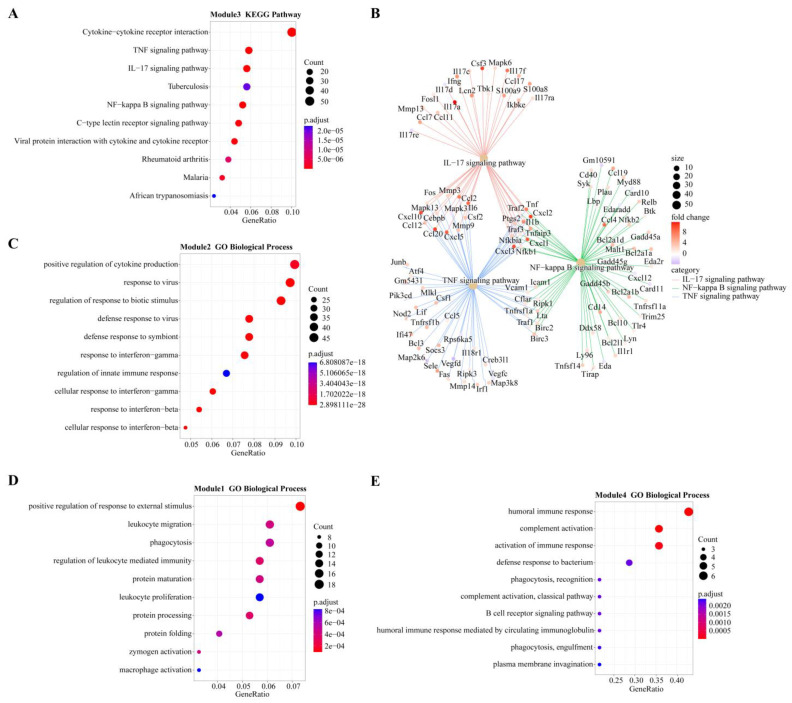
Overview of GO terms (biological process) or KEGG pathways within modules highly positively correlated with different infection stages. (**A**) KEGG pathways of module 3 (highly correlated with 12 hpi). (**B**) Genes involved in three important pathways and their expression levels at 12 hpi. Different colors represent different pathways: red for IL-17, green for NF-κB, and blue for TNF. Fold change represents the change in gene expression level compared to 0 hpi (control group). (**C**) GO terms of module 2 (highly correlated with 24 hpi). (**D**) GO terms of module 1 (highly correlated with 48 hpi). (**E**). GO terms of Module 5 (highly correlated with 96 hpi).

In module-trait analysis, module 3 was highly positively correlated with 12 hpi. GO analysis revealed that the module was mainly enriched in host immune-related biological processes, including cytokine production and mediated signaling pathways, and leukocyte migration and activation (Figure S2). KEGG analysis revealed that this module was mainly involved in immune-related and proinflammatory signaling pathways, such as the TNF, IL-17 and NF-κB signaling pathways and the C-type lectin receptor signaling pathway (Figure 5A). We also found that while the number of genes associated with the IL-17 pathway was fewer than that of the TNF and NF-κB pathways, the average upregulation multiple for the IL-17 pathway (4.9 times) was higher than that for the TNF and NF-κB pathways (3.4 and 2.6 times, respectively; Figure 5B).

Module 2 was positively correlated with 24 hpi, and GO analysis revealed it was enriched in biological processes related to the response of cytokines, including IFN-γ and IFN-β, as well as the regulation of innate immune responses to pathogens (Figure 5C). KEGG analysis revealed that the main enrichment pathways were associated with pattern recognition receptors (PRRs) (NOD-like receptors) and cytokine receptors (Figure S2).

Module 1 had the highest positive correlation with 48 hpi. According to GO analysis, this module is mainly involved in the biological process of leukocyte-related immunity and is related to the metabolic processing of protein, including protein synthesis, processing and maturation (Figure 5D). KEGG analysis also showed that pathways related to protein metabolism were enriched, including amino acid metabolism. Immune-related enrichment results included antigen processing and presentation, as well as complement and coagulation cascade pathways (Figure S2).

Modules 4, 5 and 6 were all strongly positively correlated with 96 hpi. GO analysis revealed that module 4 was enriched in biological processes related to B cells and the complement system, while module 6 was enriched in biological processes related to T cells (Figure 5E and Figure S2). Both B cell-mediated humoral immunity and T cell-mediated cellular immunity belong to adaptive immunity. The biological processes enriched in module 5 are mostly related to cell division, such as chromosome separation and DNA replication (Figure S2). KEGG analysis showed that module 5 genes were mainly involved in the pathway of the cell cycle and in T cell subset differentiation (Figure S2).

### 3.5. Cytoscape Extracts Core Genes

To further explore the key regulatory centers, we selected the gene networks with the top 200 connectivity values according to weight values from the modules (1, 2, 3, 5) with the highest positive correlation at each time point and analyzed their interactions using Cytoscape. Table 1 shows the top 20 central nodes (hub genes) in each module.

At 12 hpi, most of the genes in the top node of module 3 are closely related to the inflammatory process (*Ambp*, *Mcolon2*, *Cyp27b1*) and immune response (*Ptx3*, *plscr1*, *Dbn1*). At 24 and 48 hpi, most of the important node genes in modules 2 and 1 are related to the functional regulation of macrophages (*Rab20*, *Smpdl3b*, *Mpeg1*, *Tmem106a*, *Ccr5*, *Ms4a6d*), some of which can be stimulated by IFN *(Rab20*, *Smpdl3b*, *Mpeg1*, *Tmem106a*). This suggests that macrophages should be associated with IFN and play an indispensable role in the anti-infection process. At 96 hpi, the genes in module 5 mostly play a role in cell proliferation and cell cycle regulation (*Birc5*, *Iqgap3*, *Mbly2*, *FoxM1*, *Parpbp*).

### 3.6. Analysis of Immune Cells and PPI Network

**Figure 6:**
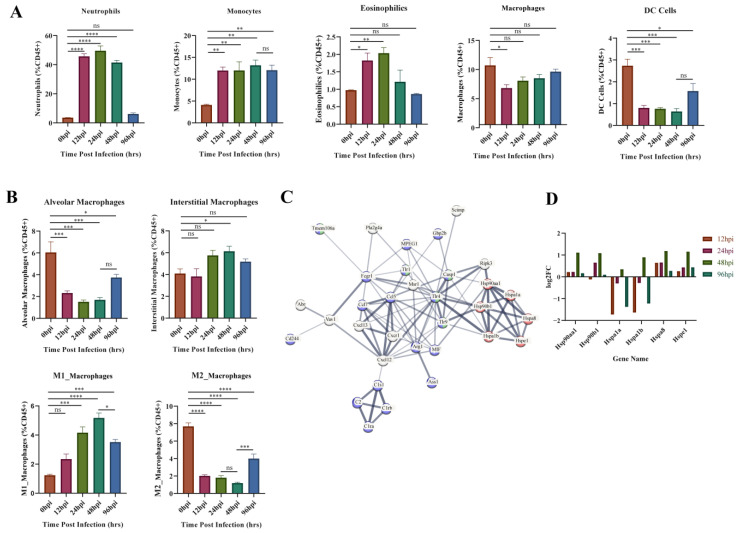
Flow cytometry and module 1 PPI network results. (**A**) Flow cytometric analysis of five major innate immune cells. (**B**) Flow cytometric analysis of four subtypes of macrophages. (**C**). Protein–protein interaction (PPI) network analysis results for module 1 genes, most associated with 48 hpi. The important genes relating to protein synthesis, macrophage activation, and innate immunity are presented. Red represents genes related to protein synthesis, blue represents genes related to innate immunity, and green represents genes related to macrophage activation. (**D**). Expression of heat shock protein genes at different time points. The ordinate indicates the number of upregulated or downregulated genes. * *p* < 0.05, ** *p* < 0.01, *** *p* < 0.001, **** *p* < 0.0001.

GO enrichment results of the upregulated gene clusters (1, 3, 4, 6) in Mfuzz identified the obvious biological processes related to cellular immunity (positive regulation of leukocyte activation, migration, and proliferation). Therefore, variation in the composition of the five types of innate immune cells throughout the infection process was investigated using flow cytometry (Figure 6A). The proportion of neutrophils, monocytes and eosinophils increased immediately after infection. Neutrophils and eosinophils peaked at 24 hpi, then fell back to the level of the control group at 96 hpi. Monocytes remained at a high level throughout the infection period. The proportion of macrophages and DC cells both decreased at 12 hpi, and DC level remained low at 12–48 hpi, while macrophages started to recover soon after 12 hpi.

Macrophage activation and protein synthesis pathways were identified from the GO enrichment results of module 1 (most associated with 48 hpi) in WGCNA analysis (Figure 5D). Therefore, we further evaluated the changes of macrophages in different subpopulations (Figure 6B). Alveolar macrophages (AM) decreased dramatically at 12–48 hpi and partially recovered at 96 hpi. Interstitial macrophages (IM) only showed a significant increase at 48 hpi. The proinflammatory M1 type rose continuously after infection until 48 hpi, after which it began to decline. In contrast, the anti-inflammatory M2-type decreases at 12–48 hpi and then begins to recover at 96 hpi. This suggests 48 hpi is a critical time point for changes in M1 versus M2 subtypes. Subsequently, all identified genes from module 1 were entered into the STRING database (https://string-db.org/, accessed on 18 November 2022) to obtain the functional protein association network. Heat shock protein (HSP)-related genes are central to the network and linked to macrophage function and innate immunity through TLRs (Figure 6C). We analyzed the expression of these HSP genes (Figure 6D) and found that *HSP90aa1*, *HSP90b1*, *HSPa8* and *HSPe1* kept upregulated at each hpi, and reached the peak at 48 hpi. In contrast, *Hspa1a* and *Hspa1b* were inhibited throughout infection but increased suddenly at 48 hpi. These phenomena indicate that the HSP family has different functions on macrophages, but that the key time point of its role is 48 hpi.

### 3.7. Quantitative RT-PCR Verification

**Figure 7:**
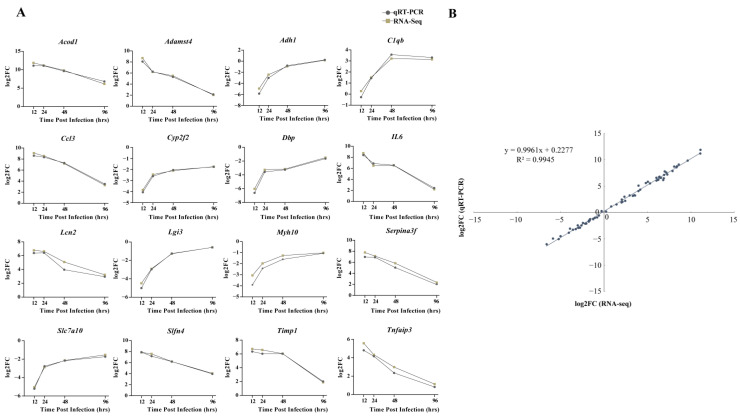
Comparison of relative expression levels measured by RNA-seq and qRT-PCR. (**A**) Sixteen differentially expressed genes (DEGs) were selected for verification. The relative expression of each gene was calculated by the 2-ΔΔCt method (fold change). The *Y*-axis shows the fold changes compared to the starting point in different stages of infection, with positive values indicating upregulation and negative values indicating downregulation. (**B**) Correlation between fold changes obtained by qRT-PCR (*X*-axis) and RNA-seq platform (*Y*-axis). Each data point was obtained from three biological replicates. Graphpad 8.0 was used to process and plot the data.

From the discovered DEGs, the following 16 genes were randomly selected for qRT-PCR detection: *Acod1*, *Adamst4*, *Adh1*, *C1qb*, *Ccl3*, *Cyp2f2*, *Dbp*, *IL6*, *Lcn2*, *Lgi3*, *Myh10*, *Serpina3f*, *Slc7a10*, *Slfn4*, *Timp1*, and *Tnfaip3*. The expression patterns of RNA-seq and qRT-PCR at four time points after infection were similar, with consistent upregulation or downregulation (Figure 7A). Moreover, a high correlation coefficient (*R^2^*) of 0.9945 confirmed the reliability of transcriptome sequencing data (Figure 7B).

## 4. Discussion

**Figure 8:**
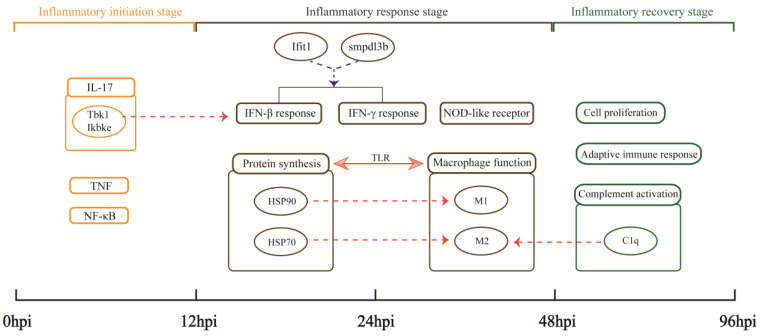
Important findings. Rectangles represent important biological processes or pathways. Circles represent important genes or the proteins they encode. Arrows indicate the existence of some relationship: the solid line shows the relationship directly discovered by the analysis in this study; the dashed lines indicate hypothesized relationships suggested by our analysis, with red indicating upregulation and blue indicating downregulation.

Pneumonia is a cause of death globally. In this study, the MRSA USA300 clone, which causes the hallmark clinical feature of human necrotizing pneumonia [31], was used to successfully construct a recovery model of severe aspiration pneumonia in mice. High-throughput RNA-seq data based on the MRSA pneumonia model to elucidate the host anti-infective process from the perspective of gene transcription were analyzed. The expression trend of DEGs and the pathological states of lungs indicate the physiological processes in mouse lungs after MRSA infection can be divided into three parts: 0–12 hpi is the inflammatory initiation stage, 12–48 hpi is the inflammatory response stage, and 48–96 hpi is the inflammatory recovery stage (Figure 8).

### 4.1. Inflammatory Initiation Stage

The number of upregulated genes was the largest at 12 hpi. This dramatic increase in expression indicates a rapid initiation of the inflammatory transcriptional response, accompanied by recruitment of innate immune cells (neutrophils, monocytes, eosinophils).

According to Mfuzz analysis, the genes (clusters 3 and 6) upregulated to peak at 12 hpi are involved in important inflammatory pathways (Figure 3). These genes enriched the biological processes of positive regulation of cytokine production and leukocyte migration. Many cytokines and chemokines (IL-6, CCL2, CCL3, CCL4, CXCL2, CXCL5, CXCL10) are significantly upregulated at 12 hpi, which can promote the recruitment of neutrophils and monocytes. Correspondingly, neutrophils and monocytes also increased significantly at 12 hpi. In contrast, the proportion of macrophages, especially alveolar macrophages, decreased at 12 hpi. This is likely due to necrotizing apoptosis induced by the USA300 toxin [32]. Studies have shown that necrotizing depletion of macrophages induced by a variety of toxins in SA amplifies the inflammatory response and is a major mechanism leading to lung injury [20]. Therefore, the absence of macrophages at the early stage indicates exacerbation of inflammation. Hence, most of the 12 hpi core genes and highly upregulated genes are involved in proinflammatory responses. *Gpr84* encodes receptor 84, the proinflammatory receptor G protein, and enhances the inflammation conducted by macrophages [33]. *Mcoln2* can encode the endolysosomal cation channel TRPML2 and play a direct role in the transport and secretion of chemokines in mouse macrophages [34]. These genes promote the further development of inflammation. The host also upregulates genes encoding antimicrobial proteins for nutrient deprivation in the inflammatory environment. *Lcn2* encodes lipocalin 2, a key antimicrobial protein that binds and sequesters bacterial siderophores [35]. *Steap4* encodes six-transmembrane epithelial antigen of the prostate 4, which has increased responsiveness to inflammatory cytokines and may increase iron and copper entry into cells and decrease circulating iron [36]. These genes work together to promote immune and inflammatory responses in MRSA pneumonia, but have not been specifically studied in MRSA pneumonia.

KEGG analysis of module 3, highly associated with 12 hpi, showed that its genes were enriched in the important inflammatory pathways IL-17, TNF, and NF-κB (Figure 5A). These pathways play important roles in host defense and disease mechanisms [37,38,39]. Moreover, we found a leading proinflammatory and immune regulatory role of the IL-17 pathway. The IL-17 family interacts with its corresponding receptors to activate downstream pathways such as NF-κB and induce the secretion of proinflammatory mediators, including IL-6, TNF-α, and IL-1β [40,41]. The *IL-17a* gene was significantly upregulated, which not only turns on inflammation, but also acts synergistically with calprotectin (encoded by *S100A8* and *S100A9*) and lipocalin 2 to protect the host during acute microbial invasion [41]. The deletion of IL-17a results in significant neutrophilic inflammation and increased mortality in mice with USA300 pneumonia [42]. In addition, the *Tbk1* and *Ikbke* genes in the IL-17 pathway respectively encode two closely related kinases: the TANK binding kinase 1 (TBK1) and the noncanonical IκB kinase (IKK-related kinase, IKKε) [43], and IFN-β can be induced by SA through the TBK1/IKKε pathway [44]. Coincidentally, IFN-β is an important result of the KEGG enrichment at 24 hpi. Therefore, we suggest that the upregulation of Tbk1 and Ikbke genes may be related to upstream initiation of host interferon mechanisms in MRSA pneumonia.

### 4.2. Inflammatory Response Stage

A strong immune inflammatory response period followed at 24 and 48 hpi. At this stage, the lesions were the most severe, while neutrophils and monocytes were continuously recruited. Moreover, IFN and macrophage mediated immune and inflammatory responses were mainly observed and potential balancing mechanisms further investigated.

SA can invade various types of nonprofessional and professional phagocytes and can survive engulfment by professional phagocytes such as macrophages [45,46]. IFN response is an important component of innate immunity to viruses and intracellular bacteria. The GO and KEGG analyses of module 2 genes, most related to 24 hpi, indicate that IFN (IFN-γ and IFN-β) response and the NOD-like receptor pathway were significantly enriched (Figure 5C). When bacteria are recognized by PRRs, including NOD-like receptors, IFN production is induced through the NF-κB pathway [47]. The secretion and mechanism of IFN in MRSA pneumonia is complex and not fully understood. IFN-γ can be secreted by NK cells and recruited neutrophils after SA lung infection, and it can promote the effective killing of MRSA by macrophages and monocytes [48,49]. However, IFN-γ also drives fatal lung inflammation during MRSA pneumonia by promoting the overproduction of inflammatory cytokines, such as TNF-α [50]. IFN-β can directly kill SA [51], but the inflammatory cascade caused by IFN-β production can also aggravate the lung damage caused by USA300 pneumonia, and the high induction of IFN-β makes it more difficult for the host to clear the bacteria in the lung [47]. Therefore, we suggest that a balanced regulation of IFN response is essential for successful defense against MRSA pneumonia. The *Ifit1* gene (significantly upregulated at 24 hpi; Figure 2D) negatively regulates the expression of TNF and many other inflammatory cytokines in macrophages stimulated by lipopolysaccharide (LPS) [52]. *Smpdl3b* (a 24 hpi core gene; Table 1) is strongly upregulated by IFN-γ stimulation and encodes a lipid-modifying enzyme, Smpdl3b, that is expressed in macrophages and acts as a negative regulator of TLR signaling at the inflammatory signaling interface [53]. The role of *ifit1* and *smpdl3b* in host immune mechanisms against MRSA pneumonia has not been investigated. We hypothesize that these genes regulate the balance between inflammation and IFN bactericidal action to promote optimal innate immunity.

Transcriptome analysis showed that macrophage activation is associated with pathways involved in protein synthesis and is highly correlated with HSP70 and HSP90 genes (Figure 5D and Figure 6C). These results are similar to those found in proteomic analysis of macrophages after LPS stimulation [54]. Macrophages are plastic immune cells necessary to maintain homeostasis and control inflammation [55]. M1-type (proinflammatory) macrophages began to rise immediately after infection and remain at a high level until 24–48 hpi (Figure 6B). *Hsp90aa1* and *Hsp90b1* genes are increased in the inflammatory environment (Figure 6D), and the encoded HSP90 can promote macrophage-mediated inflammation [56]. More recently, it has been shown that HSP90 binds to SA peptides to ward off the deadly infection of SA bacteremia, possibly in connection with a faster or stronger innate immune response to SA infections [57]. Taken together with our study results, we propose that HSP90 may promote the polarization of M1-type macrophages and play a positive role in defense against MRSA pneumonia. Interestingly, HSP70s genes showed two trends: HSPa8 expression increased at 12–48 hpi, while Hspa1a and Hspa1b expression decreased at 12–24 hpi and increased only at 48 hpi (Figure 6D). HSP70s have anti-inflammatory effects. In macrophages, high expression of both HSC70 (*HSPa8* encoding) and HSP70 (*Hspa1a* and *Hspa1b* encoding) inhibits the expression of LPS-induced inflammatory factors by inactivating NF-κB [58,59]. Meanwhile, the proportion of M2-type macrophages (anti-inflammatory) decreased at 12–48 hpi and partially recovered after that (Figure 6B). Therefore, we speculate that the increased expression of HSP70 may be a mechanism to prevent the decrease of M2-type macrophages and inhibit excessive inflammation. Likewise, *Tmem106a* (core gene at 48 hpi) plays a negative regulatory role in macrophage-mediated inflammation, and deletion of this gene may lead to enhanced M1-type polarization of macrophages [60]. Together, these results indicate that although the proinflammatory response was still dominant in the inflammatory response stage, the anti-inflammatory response also began to be enhanced at this stage.

### 4.3. Inflammatory Recovery Stage

The fewest DEGs were present at 96 hpi, when most of the lesions in the lungs had recovered. Cluster 1 genes, enriched in the biological processes of cell division and adaptive immune response in the GO enrichment results, whose expression was still upregulated at 96 hpi (Figure 3). Cluster 3, 4 and 6 genes, significantly enriched in the biological processes of cytokine production and leukocyte migration, mostly returned to expression levels close to those at 0 hpi (Figure 3). This suggests that transcription of lung genes was no longer predisposed to recruit large numbers of cells, making the proportion of neutrophils and eosinophils similar to that at 0 hpi. Interestingly, the proportion of monocytes remained at a high level (Figure 6A). Monocytes can transform and supplement the consumption of macrophages to ensure the normal immune function of the body [61]. M1-type macrophages began to decline and return toward values observed at 0 hpi. M2-type macrophages increased at 96 hpi compared to 12–48 hpi (Figure 6B). Changes in cell composition validated reversal of host inflammatory tendencies.

There are many related gene modules of 96 hpi, which indicates the diversity of genes in the recovery state. The process of cell proliferation, complement activation and adaptive immune response can be found from the GO and KEGG analysis results (Figure 5E and Figure S2), respectively. The complement system is an essential component of immunity. *C1qb, C1qc* and *C1qa*, which correlate with complement factor C1q, were observed in significantly upregulated genes of 96 hpi (Figure 2D). C1q is important for initiating the classical complement pathway. Its importance in the defense against *Streptococcus pneumoniae* infection has been well established [62,63,64]. Moreover, C1q is mainly produced by macrophages and can induce the M2 polarization of macrophages [65]. Therefore, it may be another cause of subtype differentiation of macrophages in MRSA pneumonia. In addition, it is now widely believed that the complement system controls T cell and B cell responses, cross-linking innate and adaptive immunity [66,67]. However, the mechanism of the complement system including C1q in resisting MRSA pneumonia has not been clarified, which is worth further exploration.

## 5. Conclusions

In this study, the resolution of pulmonary MRSA infection and the restoration of homeostasis involved finely regulated behavior between proinflammatory and anti-inflammatory responses. Given the correlation between cell transformation and key proteins in immune mechanisms illustrated in this study, further exploration using single-cell transcriptomics and proteomic techniques is warranted. This study provides new insights into the immune mechanisms underlying MRSA pneumonia, indicating new directions for research to find a nonantibiotic treatment.

## Figures and Tables

**Table 1:** Top 20 hub nodes in the progression-related modules were identified by the gene modules in the weighted gene coexpression network analysis (WGCNA).

Module 1		Module 2		Module 3		Module 5	
Gene	Dgree	Gene	Dgree	Gene	Dgree	Gene	Dgree
Mpeg1	23	Rab20	24	Ambp	36	C330027C09Rik	30
Eno1	22	Smpdl3b	21	Plscr1	20	Birc5	30
Tmem106a	21	Ccl3	17	Mcoln2	17	Tpx2	29
X4930430E12Rik	19	Igtp	14	Dbn1	16	Iqgap3	16
Ms4a6c	19	Gbp2	14	Ptx3	16	Mybl2	12
Ccr5	18	Ifi47	14	Sdc4	16	Foxm1	12
Gba	16	Wars	13	Cyp27b1	13	Parpbp	12
Fcgr1	15	Gbp11	13	Ccl17	13	Kif22	12
Ms4a6d	13	Rnd3	12	Il1bos	10	Ncapg	11
Gla	12	Parp12	11	Adm	10	Kifc1	10
Slc7a8	11	F10	11	Rdh12	8	Cep55	10
Tlr9	10	Cxcl9	10	Alx4	8	Top2a	9
Tgfbi	10	Sod2	10	Gm45774	8	Bub1b	9
Cln3	10	Nos2	9	Calcr	7	Kif15	8
Fbn1	9	Cd274	8	Ddn	7	Nuf2	8
AB124611	9	Gbp4	8	Ngp	6	Gm38411	7
Bpifb5	8	Snx10	8	Hdx	6	Tcrg.C1	7
Gm15931	7	Tgtp1	7	Avpr1a	6	Trdc	7
Scimp	7	Batf2	6	Inhbb	6	Gm43434	7
Hsp90b1	6	Fam26e	6	Trem1	5	Apol7b	7

## Data Availability

Raw reads were deposited in the Gene Expression Omnibus (GEO) repository (accession number GSE220943). The original contributions presented in the study are publicly available. These data can be found at https://www.ncbi.nlm.nih.gov/geo/query/acc.cgi?acc=GSE220943, accessed on 15 April 2023.

## Supplementary Materials

The following supporting information can be downloaded at https://www.mdpi.com/article/10.3390/biom13020347/s1. Figure S1: The specific cell classification scheme is shown in the sample flow cytometry results of 0 hpi; Figure S2: Overview of GO terms (biological process) or KEGG pathways within modules 1–6; Table S1: Primer sequences used for qRT-PCR in this study.
