# Supplementary material for: Time-Course Transcriptome Analysis of the Lungs of Mice Challenged with Aerosols of Methicillin-Resistant Staphylococcus aureus USA300 Clone Reveals Inflammatory Balance
Source: Biomolecules. 2023 Feb 10;13(2):347. doi: 10.3390/biom13020347 (PMC9953551; doi:10.3390/biom13020347)
Supplement: Supplementary file 1 [file biomolecules-13-00347-s001.zip › Figure S1 and Figure S2_correct version.pdf]

1.1 Supplementary Figures

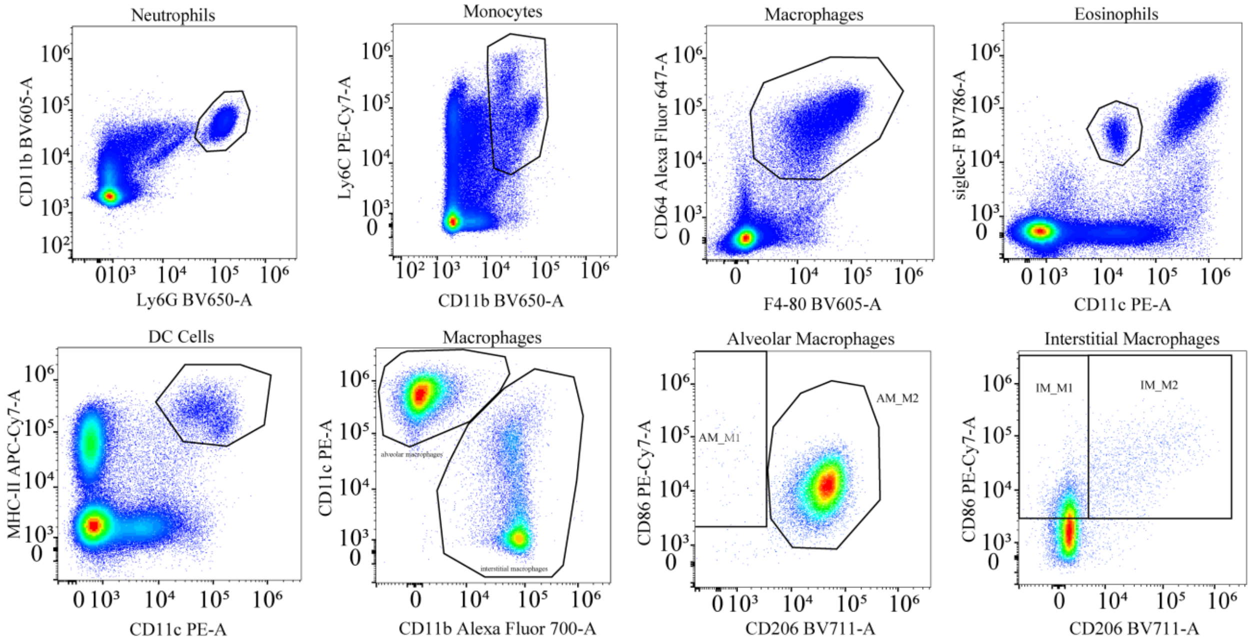

Figure S1. The specific cell classification scheme is shown in the sample flow cytometry results of 0 hpi.

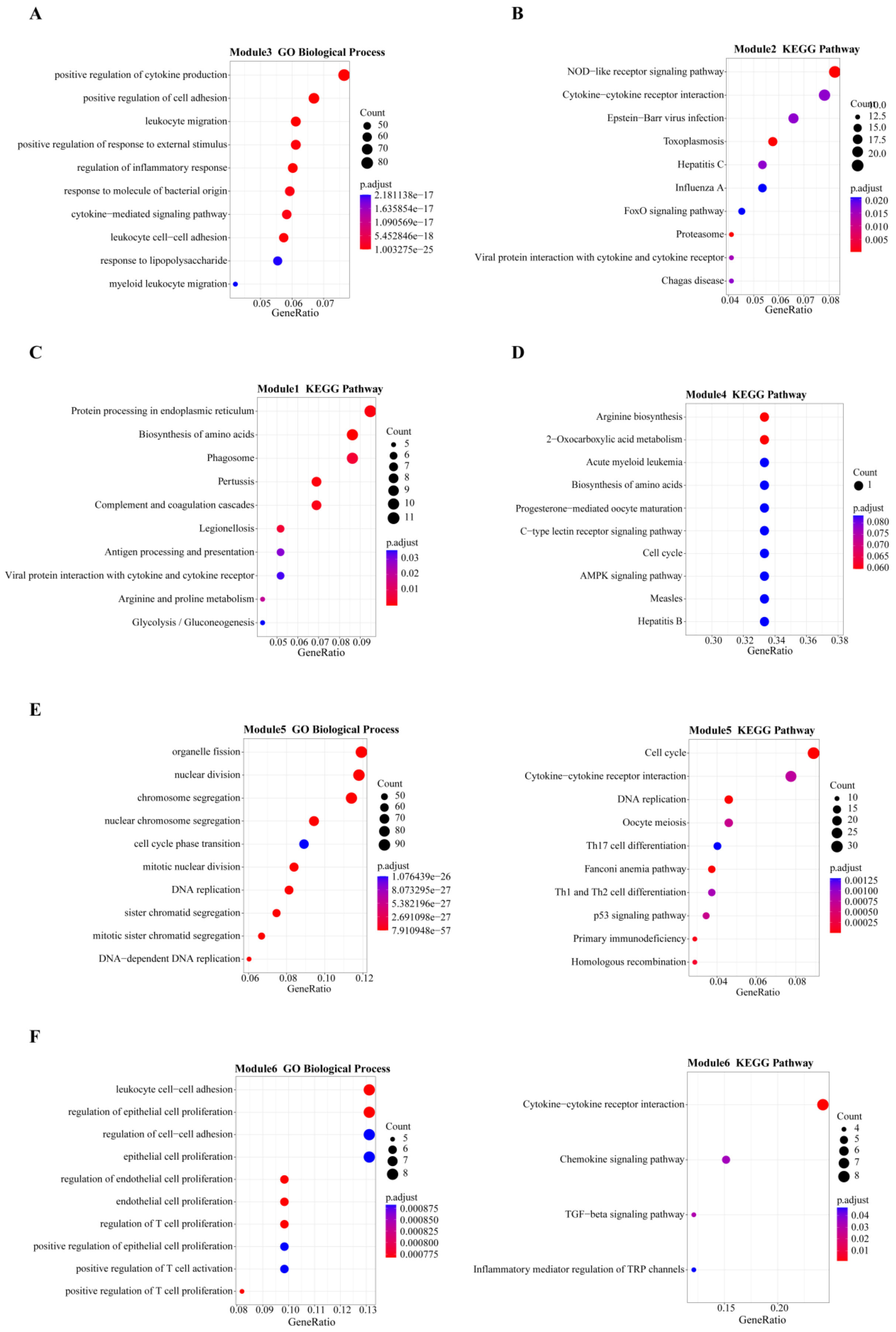
A
Module3 GO Biological Process
positive regulation of cytokine production
positive regulation of cell adhesion
leukocyte migration
positive regulation of response to external stimulus
regulation of inflammatory response
response to molecule of bacterial origin
cytokine−mediated signaling pathway
leukocyte cell−cell adhesion
response to lipopolysaccharide
myeloid leukocyte migration
0.05 0.06 0.07
GeneRatio
Count
50
60
70
80
p.adjust
2.181138e−17
1.635854e−17
1.090569e−17
5.452846e−18
1.003275e−25
B
Module2 KEGG Pathway
NOD−like receptor signaling pathway
Cytokine−cytokine receptor interaction
Epstein−Barr virus infection
Toxoplasmosis
Hepatitis C
Influenza A
FoxO signaling pathway
Proteasome
Viral protein interaction with cytokine and cytokine receptor
Chagas disease
0.04 0.05 0.06 0.07 0.08
GeneRatio
Count
10.0
12.5
15.0
17.5
20.0
p.adjust
0.020
0.015
0.010
0.005
C
Module1 KEGG Pathway
Protein processing in endoplasmic reticulum
Biosynthesis of amino acids
Phagosome
Pertussis
Complement and coagulation cascades
Legionellosis
Antigen processing and presentation
Viral protein interaction with cytokine and cytokine receptor
Arginine and proline metabolism
Glycolysis / Gluconeogenesis
0.05 0.06 0.07 0.08 0.09
GeneRatio
Count
5
6
7
8
9
10
11
p.adjust
0.03
0.02
0.01
D
Module4 KEGG Pathway
Arginine biosynthesis
2−Oxocarboxylic acid metabolism
Acute myeloid leukemia
Biosynthesis of amino acids
Progesterone−mediated oocyte maturation
C−type lectin receptor signaling pathway
Cell cycle
AMPK signaling pathway
Measles
Hepatitis B
0.30 0.32 0.34 0.36 0.38
GeneRatio
Count
1
p.adjust
0.080
0.075
0.070
0.065
0.060
E
Module5 GO Biological Process
organelle fission
nuclear division
chromosome segregation
nuclear chromosome segregation
cell cycle phase transition
mitotic nuclear division
DNA replication
sister chromatid segregation
mitotic sister chromatid segregation
DNA−dependent DNA replication
0.06 0.08 0.10 0.12
GeneRatio
Count
50
60
70
80
90
p.adjust
1.076439e−26
8.073295e−27
5.382196e−27
2.691098e−27
7.910948e−57
Module5 KEGG Pathway
Cell cycle
Cytokine−cytokine receptor interaction
DNA replication
Oocyte meiosis
Th17 cell differentiation
Fanconi anemia pathway
Th1 and Th2 cell differentiation
p53 signaling pathway
Primary immunodeficiency
Homologous recombination
0.04 0.06 0.08
GeneRatio
Count
10
15
20
25
30
p.adjust
0.00125
0.00100
0.00075
0.00050
0.00025
F
Module6 GO Biological Process
leukocyte cell−cell adhesion
regulation of epithelial cell proliferation
regulation of cell−cell adhesion
epithelial cell proliferation
regulation of endothelial cell proliferation
endothelial cell proliferation
regulation of T cell proliferation
positive regulation of epithelial cell proliferation
positive regulation of T cell activation
positive regulation of T cell proliferation
0.08 0.09 0.10 0.11 0.12 0.13
GeneRatio
Count
5
6
7
8
p.adjust
0.000875
0.000850
0.000825
0.000800
0.000775
Module6 KEGG Pathway
Cytokine−cytokine receptor interaction
Chemokine signaling pathway
TGF−beta signaling pathway
Inflammatory mediator regulation of TRP channels
0.15 0.20
GeneRatio
Count
4
5
6
7
8
p.adjust
0.04
0.03
0.02
0.01

Figure S2. Overview of GO terms (biological process) or KEGG pathways within modules 1-6. **A**. GO terms of Module 3 (highly correlated with 12 hpi). **B.** KEGG pathways of Module 2 (highly correlated with 24 hpi). **C.** KEGG pathways of Module 1 (highly correlated with 48 hpi). **D.** KEGG pathways of Module 4. **B.** GO terms and KEGG pathways of Module 5. **B.** GO terms and KEGG pathways of Module 6. Module 4, 5 and 6 are highly correlated with 96 hpi.
